# Supplementary material for: Dynamic localization of the chromosomal passenger complex in trypanosomes is controlled by the orphan kinesins KIN-A and KIN-B
Source: eLife. 2024 Apr 2;13:RP93522. doi: 10.7554/eLife.93522 (PMC10987093; doi:10.7554/eLife.93522)
Supplement: Figure 2—figure supplement 1—source data 2. [file elife-93522-fig2-figsupp1-data2.zip › Figure 2ΓÇôfigure supplement 1ΓÇôsource data 2/Figure_2ΓÇôfigure_supplement_1_Source_data_2.pdf]

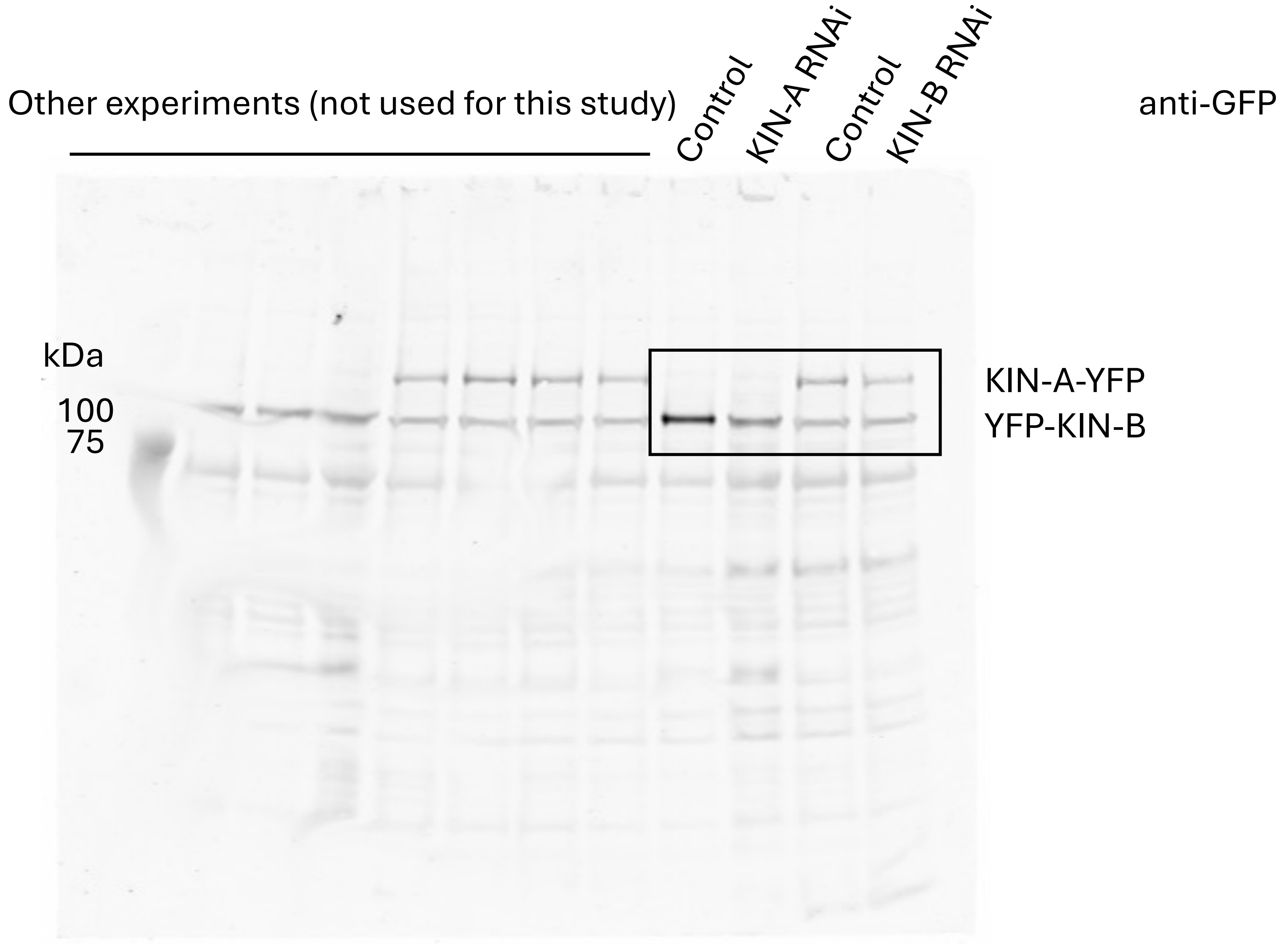
Other experiments (not used for this study)
Control
KIN-A RNAi
Control
KIN-B RNAi
anti-GFP
kDa
100
75
KIN-A-YFP
YFP-KIN-B

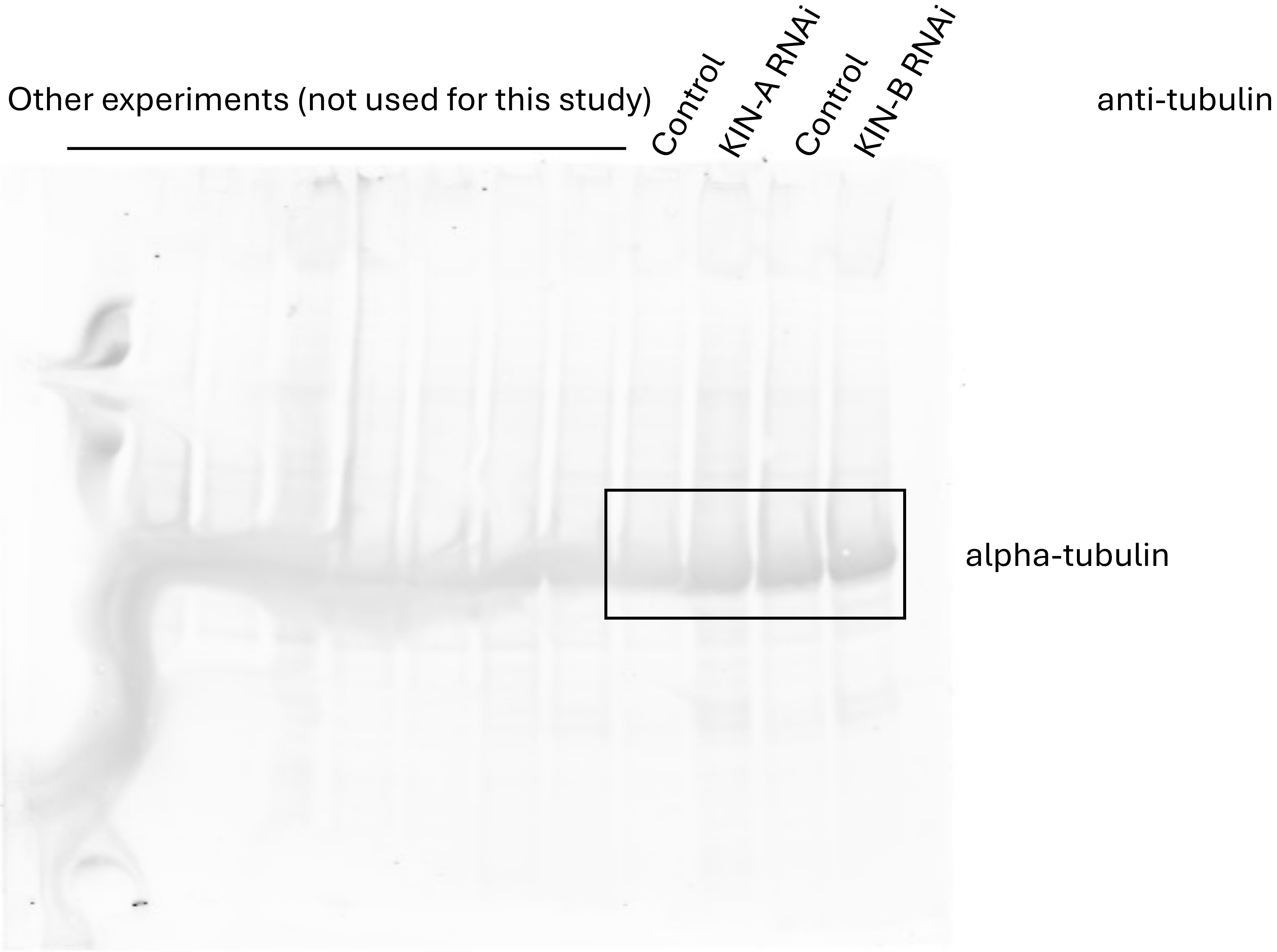

Other experiments (not used for this study)
Control
KIN-A RNAi
Control
KIN-B RNAi
anti-tubulin
alpha-tubulin
