# Supplementary figures and images for: Dynamic localization of the chromosomal passenger complex in trypanosomes is controlled by the orphan kinesins KIN-A and KIN-B
Source: eLife. 2024 Apr 2;13:RP93522. doi: 10.7554/eLife.93522 (PMC10987093; doi:10.7554/eLife.93522)

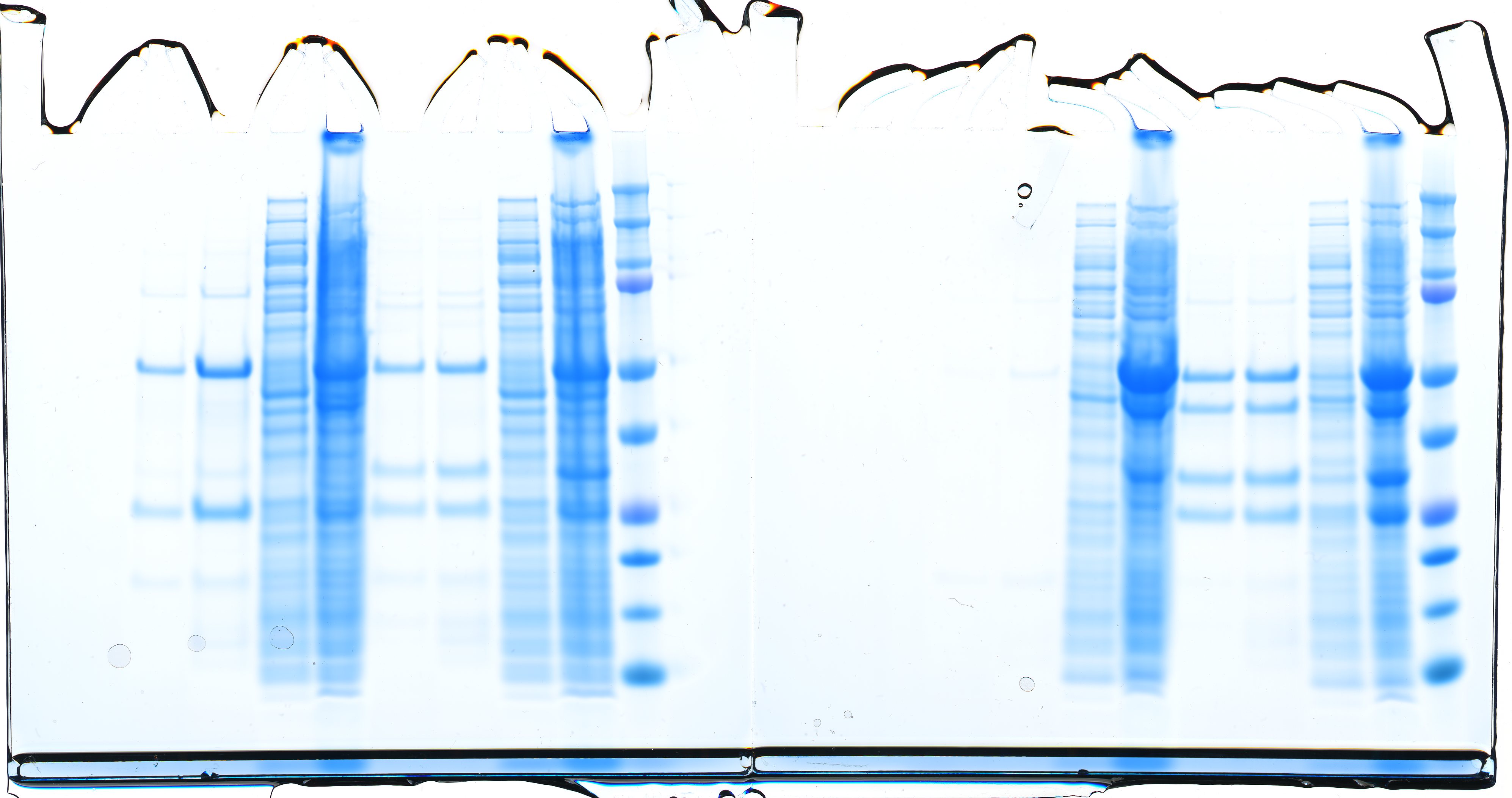

Supplement: Figure 4—source data 1. [file elife-93522-fig4-data1.zip › Figure 4ΓÇôsource data 1/Figure_4_Source_data_1.jpg]

plasmids transformed  
into *E. coli*:

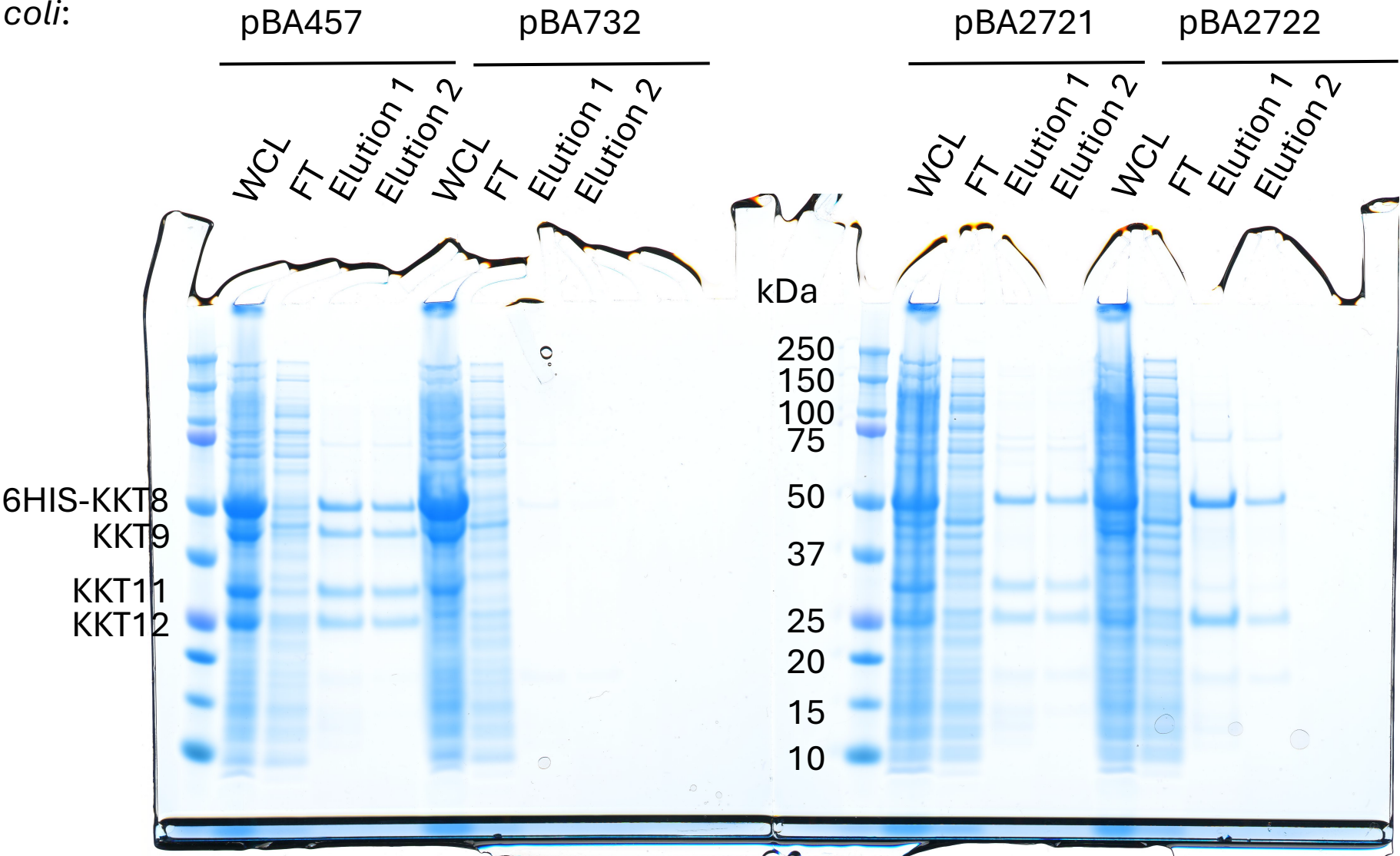

Supplement: Figure 4—source data 2. [file elife-93522-fig4-data2.zip › Figure 4ΓÇôsource data 2/Figure_4_Source_data_2.pdf]

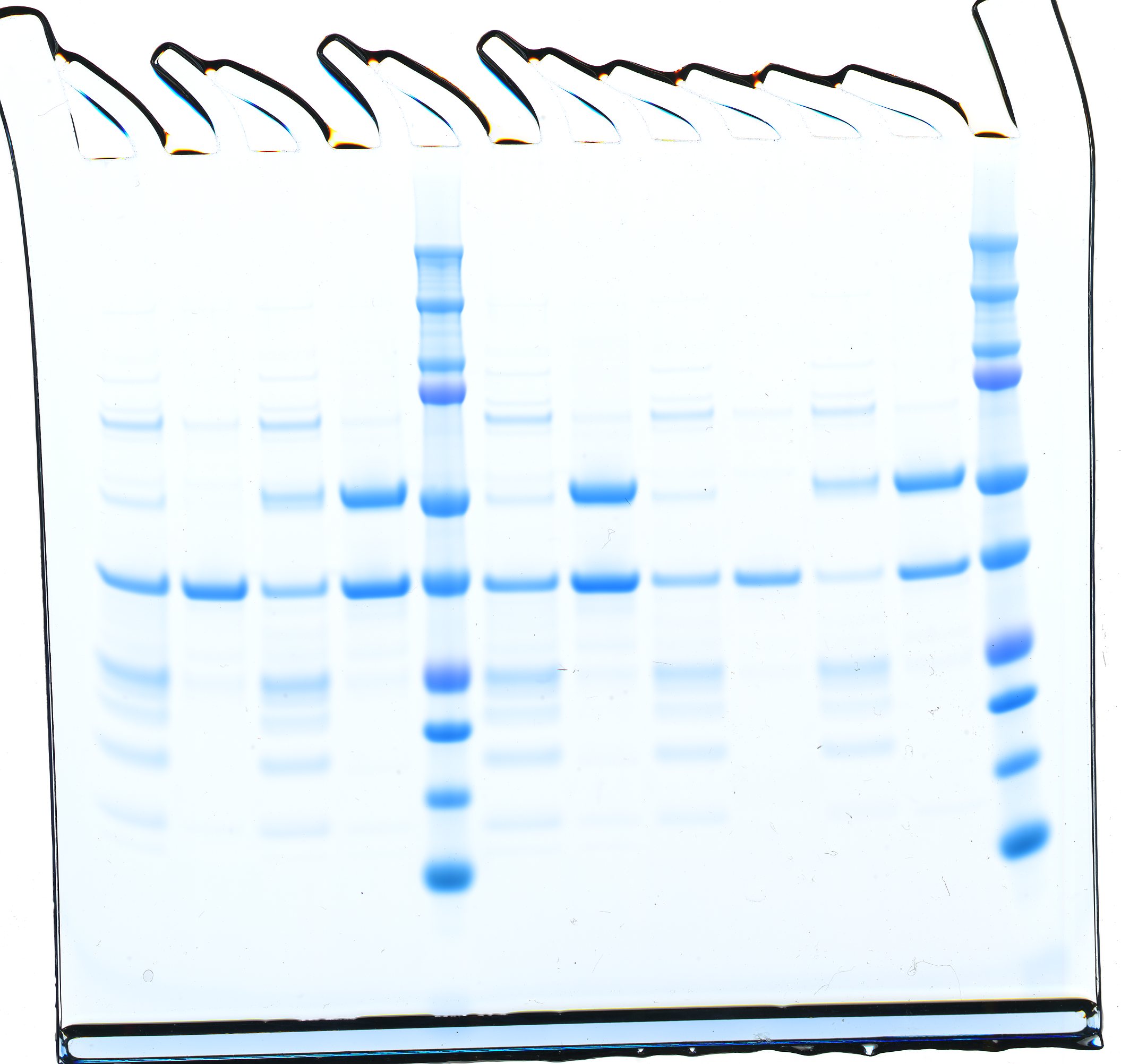

Supplement: Figure 6—figure supplement 2—source data 1. [file elife-93522-fig6-figsupp2-data1.zip › Figure 6ΓÇôfigure supplement 2ΓÇôsource data 1/Figure_6ΓÇôfigure_supplement_2A_raw_KIN-A_MTcosedimentation.jpg]

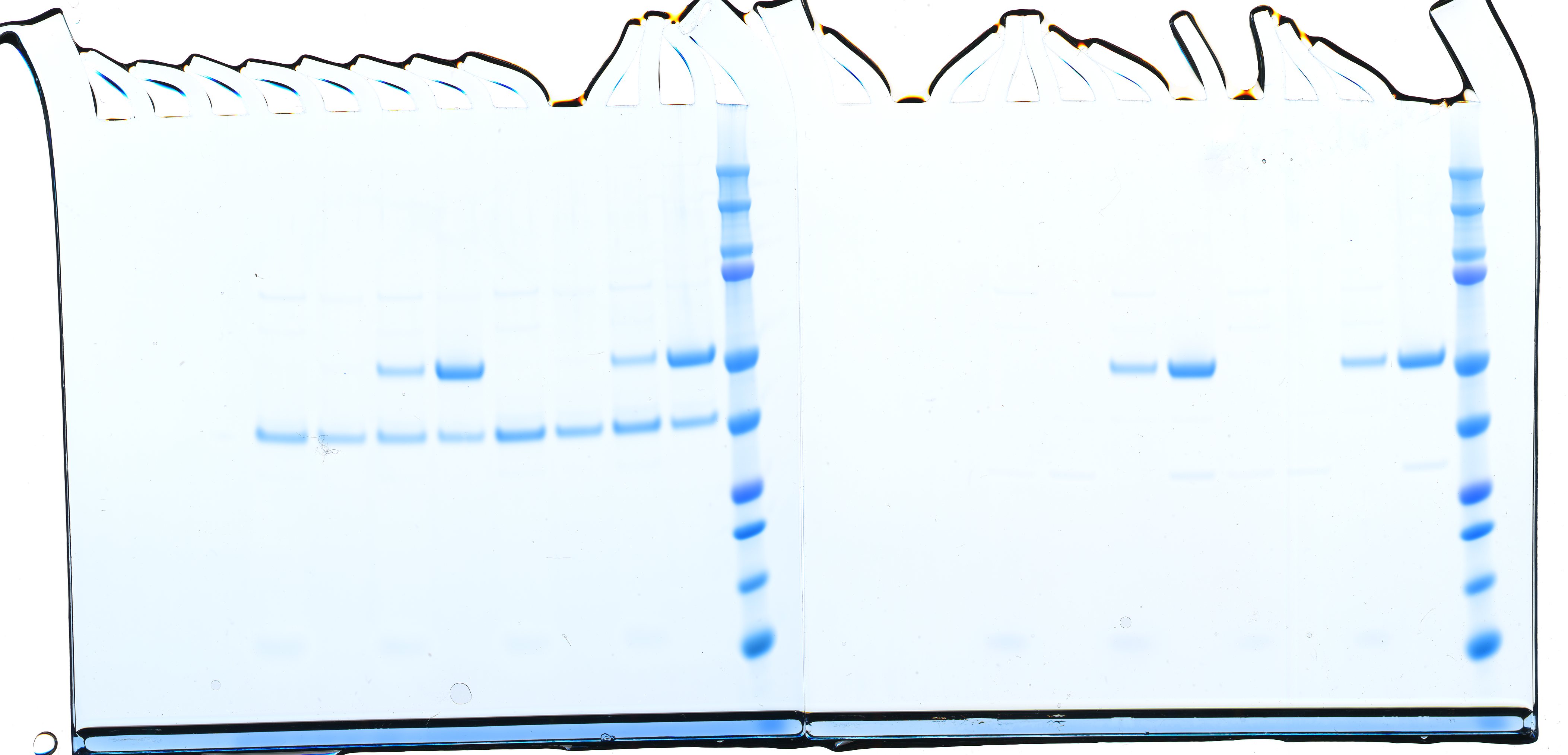

Supplement: Figure 6—figure supplement 2—source data 1. [file elife-93522-fig6-figsupp2-data1.zip › Figure 6ΓÇôfigure supplement 2ΓÇôsource data 1/Figure_6ΓÇôfigure_supplement_2A_raw_KIN-B_MTcosedimentation.jpg]

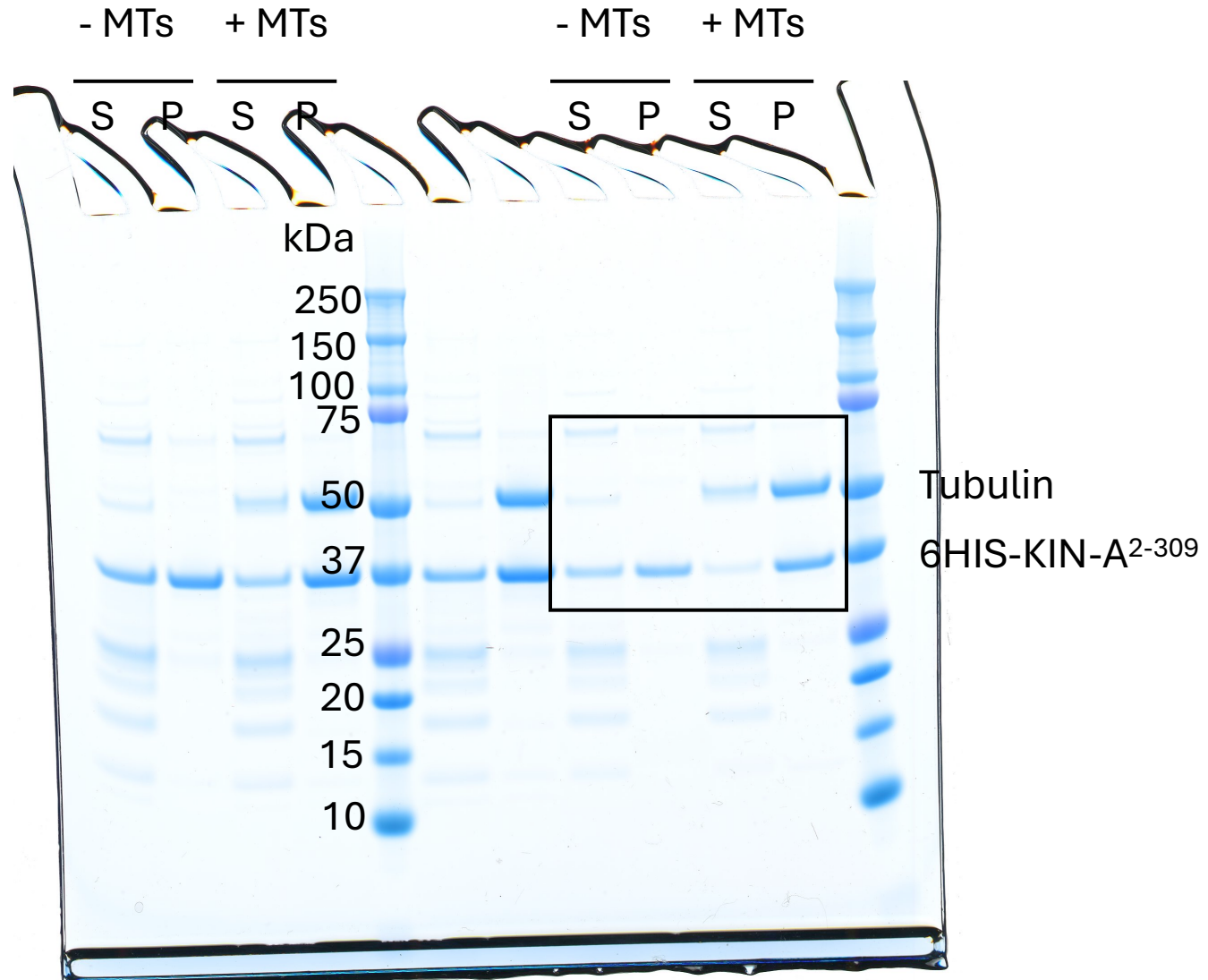

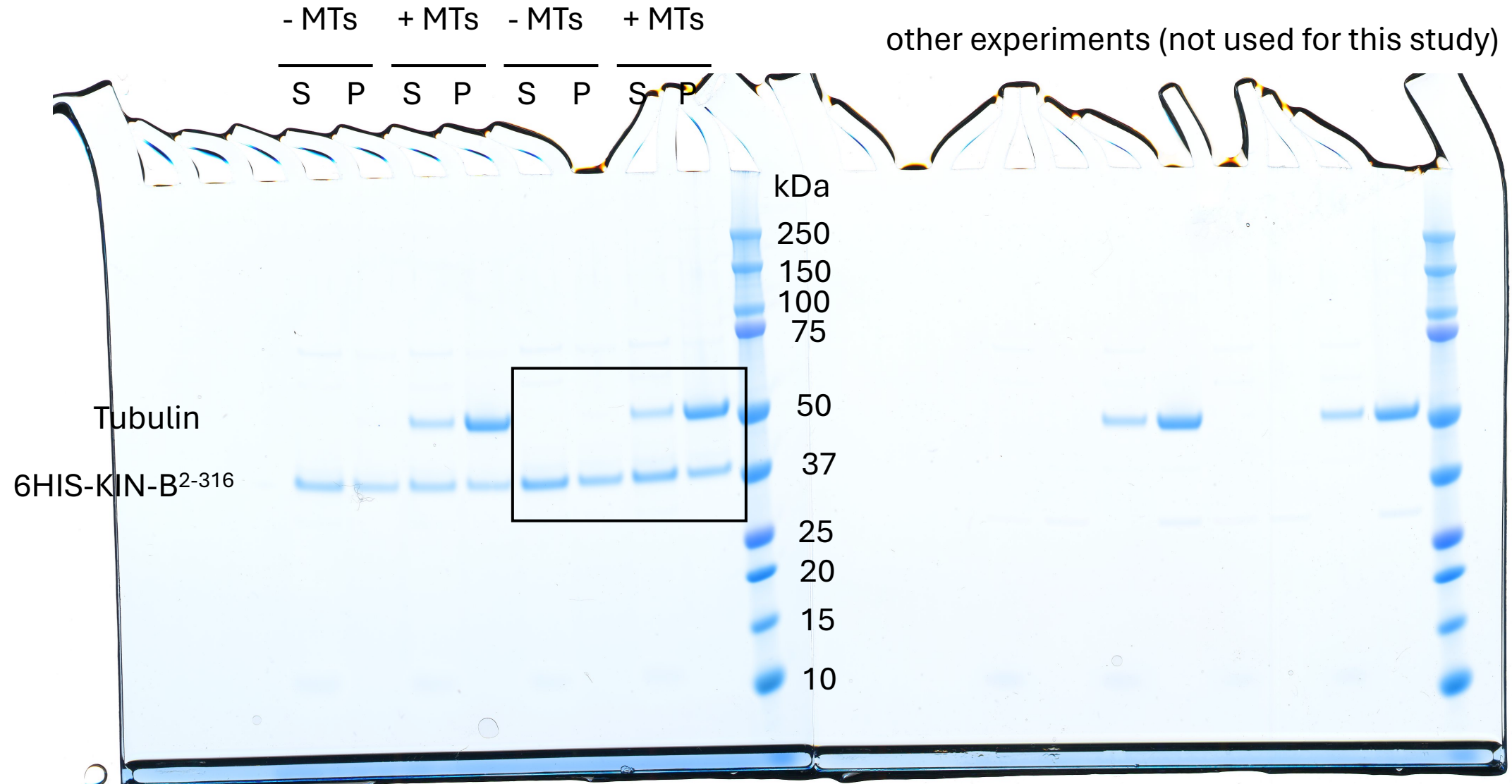

Supplement: Figure 6—figure supplement 2—source data 2. [file elife-93522-fig6-figsupp2-data2.zip › Figure 6ΓÇôfigure supplement 2ΓÇôsource data 2/Figure_6ΓÇôfigure_supplement_2_Source_data_2.pdf]
